# Using Machine Learning to Predict 30-Day Hospital Readmissions in Patients with Atrial Fibrillation Undergoing Catheter Ablation

**DOI:** 10.3390/jpm10030082

**Published:** 2020-08-09

**Authors:** Man Hung, Evelyn Lauren, Eric Hon, Julie Xu, Bianca Ruiz-Negrón, Megan Rosales, Wei Li, Tanner Barton, Jacob O’Brien, Weicong Su

**Affiliations:** 1 College of Dental Medicine, Roseman University of Health Sciences, South Jordan, UT 84095, USA; 2 Department of Biostatistics, Boston University, Boston, MA 02218, USA; evelyn.lauren@utah.edu; 3 Department of Economics, University of Chicago, Chicago, IL 60637, USA; ericstephenhon@uchicago.edu; 4 College of Nursing, University of Utah, Salt Lake City, UT 84112, USA; julie.xu@utah.edu; 5 School of Medicine, University of Utah, Salt Lake City, UT 84032, USA; bianca.ruiznegron@utah.edu; 6 Division of Public Health, University of Utah, Salt Lake City, UT 84108, USA; meg.rosales@utah.edu; 7 Office of the Utah Legislative Auditor General, Salt Lake City, UT 84114, USA; li.wei@utah.edu; 8 School of Business, University of Utah, Salt Lake City, UT 84112, USA; tanner.barton@utah.edu; 9 College of Dental Medicine, Roseman University of Health Sciences, South Jordan, UT 84095, USA; jobrien1@student.roseman.edu; 10 Department of Mathematics, University of Utah, Salt Lake City, UT 84112, USA; sochachai0880@gmail.com

**Keywords:** atrial fibrillation, machine learning, artificial intelligence, hospital readmissions, heart, NRD, catheter ablation, quality improvement, risk modeling, clinical outcome

## Abstract

Atrial fibrillation (AF) cases are expected to increase over the next several decades, due to the rise in the elderly population. One promising treatment option for AF is catheter ablation, which is increasing in use. We investigated the hospital readmissions data for AF patients undergoing catheter ablation, and used machine learning models to explore the risk factors behind these readmissions. We analyzed data from the 2013 Nationwide Readmissions Database on cases with AF, and determined the relative importance of factors in predicting 30-day readmissions for AF with catheter ablation. Various machine learning methods, such as *k*-nearest neighbors, decision tree, and support vector machine were utilized to develop predictive models with their accuracy, precision, sensitivity, specificity, and area under the curve computed and compared. We found that the most important variables in predicting 30-day hospital readmissions in patients with AF undergoing catheter ablation were the age of the patient, the total number of discharges from a hospital, and the number of diagnoses on the patient’s record, among others. Out of the methods used, *k*-nearest neighbor had the highest prediction accuracy of 85%, closely followed by decision tree, while support vector machine was less desirable for these data. Hospital readmissions for AF with catheter ablation can be predicted with relatively high accuracy, utilizing machine learning methods. As patient age, the total number of hospital discharges, and the total number of patient diagnoses increase, the risk of hospital readmissions increases.

## 1. Introduction

Atrial fibrillation (AF) is a costly, widespread, and steadily growing comorbidity. Known as the most common sustained cardiac rhythm disorder [1], it is estimated to affect 33.5 million individuals globally [2], with the number of affected individuals projected to increase exponentially over the next four decades [3]. In the United States (US), the number of AF cases is expected to increase at least two-fold by 2050 [4]. The projected, rapid increase in the number of cases is attributed to the rise in the elderly population around the globe [3], as AF is closely related to the aging process [5]. Currently, the rise in AF cases corresponds to an increase in medical costs, contributing to the public health crisis. The total annual medical cost for atrial fibrillation treatments in the US was estimated at $6.65 billion in 2006 [6], and is expected to increase rapidly alongside the aging population.

AF is defined as rapid, irregular, and chaotic electrical activity in the atria, causing symptoms such as palpitations, shortness of breath, effort intolerance and fatigue [2], and is related to an increase in morbidity and mortality rate, from heart failure, stroke, cognitive impairment [7], and other thromboembolic complications [8]. These symptoms have resulted in AF patients having a significantly lower quality of life compared to the general population and other patients with coronary heart diseases [1,9]. A well-established treatment option for atrial fibrillation that is increasing in popularity is catheter ablation [10]. The use of radiofrequency or cryotherapy to electrically isolate the pulmonary veins and ablate arrhythmia foci [11] during catheter ablation can result in the improvement of atrial fibrillation-related symptoms and an increase in health-related quality of life (HQoL) [2]. Ablation is also observed to lower the risk of death, stroke, and dementia [8], and is more effective in relieving symptoms compared to the usage of anti-arrhythmic medications [12].

In an effort to improve the quality of healthcare while simultaneously reducing healthcare costs, the US Centers for Medicaid and Medicare Services have developed the Hospital Readmission Reduction Program (HRRP), which penalizes healthcare providers and entities for high readmission rates [13,14]. The implementation of HRRP has shown to be successful in reducing readmission rates by about 1% [15]. However, with 2592 out of 5627 US hospitals penalized in 2015, the overall readmission rate in the US is still high [13]. Heart attack and heart failure are among the predominant hospitalization diagnoses affected by the penalty imposed by HRRP, and are conditions that are heavily comorbid with AF. Understanding the reasons behind hospital readmissions in AF patients is critical for reducing HRRP penalties and minimizing the rising healthcare costs that can be incurred due to the rise in AF cases.

The 30-day hospital readmission rate for AF patients undergoing catheter ablation is around 10%, due to reasons such as atrial fibrillation, atrial flutter, and procedural complications [11]. Age, sex, primary payer, heart failure, hypertension, chronic renal disease, lung disease, and the number of AF hospitalizations during the prior year were significant univariate predictors for 30-day hospital readmittance [11]. Although readmission rates for AF patients (10%) [16] are lower compared to those for other conditions affected by HRRP penalties, such as acute myocardial infarction (20%), heart failure (25%), and pneumonia (18%) [17], they are still significant. Compared to the general population, AF patients are three times more likely to undergo multiple hospitalizations and spend 73% more annually in direct medical costs, which include Medicare payments [1,18]. As AF cases are expected to increase within the next few decades, so is the urgency to understand AF, in order to alleviate the impending economic and public health burden.

Past research pertaining to hospital readmissions have typically used traditional hypothesis-driven statistical techniques to identify the causal factors, which rely heavily on assumptions, and are riddled with limitations when data are expanded to include a large range of variables [19,20]. Hospital readmission data are typically derived from a huge database with a large number of variables, and are susceptible to the limitations imposed by traditional hypothesis-driven techniques. Machine learning is an innovative approach that allows a large amount of data to be processed efficiently without relying on traditional assumptions, and allows the creation of models tailored to individual patient treatment. The focus of this study was to use data-driven techniques to create better prediction models of the 30-day readmissions for AF patients undergoing catheter ablation.

## 2. Materials and Methods

### 2.1. Data

This study used data from the 2013 cycle of the Nationwide Readmissions Database (NRD). The NRD is part of a family of databases developed for the Healthcare Cost and Utilization Project (HCUP), and addresses the lack of nationally representative information on hospital readmissions for all ages. The NRD uses HCUP State Inpatient Databases (SID) and corresponding verified patient numbers to track patients within selected states, while adhering to strict privacy guidelines. The target population was limited to inpatient discharges treated at community hospitals that were not rehabilitation or long-term acute care facilities. The 2013 NRD was constructed from 21 SID that contained geographically dispersed information, and comprised 49.3% of the total US population and 49.1% of all US hospitalizations. Additional details regarding NRD can be found online at www.hcup-us.ahrq.gov/nrdoverview.jsp [21].

### 2.2. Outcome

The primary outcome for this study was the 30-day readmissions status. The NRD defined an index event as the starting point for analyzing repeat hospital visits, while hospital readmission was defined as a subsequent inpatient admission within a specified time period. Subsequently, 30-day readmissions were defined as the index admissions that had at least one readmission within the 30 days after hospital discharge.

### 2.3. Demographics

The demographic variables used in this study included age, number of unique chronic conditions, diagnosis, and procedures reported for a patient on their discharge, patients’ length of hospital stay, gender, income, and primary payer. Both weighted and unweighted prevalence estimates were calculated for the demographics. To compute the weighted demographic descriptive statistics, the R 4.0.0 survey package was used. Clusters, stratum, and weights were incorporated into the data to obtain nationally representative results. Due to certain population subgroups having small or disproportionate sample sizes, the application of sampling weights enables a sufficient sample size for statistical analyses, and leads to enhanced precision. To calculate the weighted estimates, the NRD raw data are multiplied by the sampling weights. The incorporation of sampling weights makes it possible for converting the NRD raw data collected from a sample of the US’ population to nationally representative population estimates in all 50 states in the US. Sampling weights represent selection probability of the samples and are used to adjust systematic differences or biases in probability sampling, so that the results derived from the study are reflective of the national population.

### 2.4. Data Processing

Using the International Classification of Diseases, Ninth Revision, Clinical Modification (ICD-9-CM), patients were identified with the diagnosis code for AF (427.31) as the primary diagnosis and the procedural code for catheter ablation (37.34), as the primary or secondary procedure. Patients under the age of 18 years old, who died during hospitalization, or had a missing length of stay, were excluded. For the 30-day readmission status, patients discharged after November were excluded to account for the 30-day follow-up. Cases with the following secondary diagnoses were excluded: atrial flutter, paroxysmal supraventricular tachycardia, atrioventricular (AV) nodal tachycardia, Wolff–Parkinson–White syndrome, paroxysmal ventricular tachycardia, and ventricular premature beats [11,22]. Additional exclusion criteria were cases with diagnoses or procedural codes showing prior or current implantation of a pacemaker or implantable cardioverter-defibrillator and cases with open surgical ablations [11,22].

Further data processing was conducted, in order to prepare the data for variable selection. Non-predictor variables, such as patient IDs, key identifiers, and weighting variables were excluded. Variables with all cases missing were dropped. Additionally, age and total hospital discharges were standardized so that the scales were consistent with all other variables. To prepare the data for machine learning classification, resampling methods were applied to the readmitted cases, in order to account for the imbalanced data. Categorical data (hospital bed size, discharge quarter, etc.) were dummy coded, to avoid the classifiers from incorrectly interpreting the variables as continuous data.

### 2.5. Variable Selection

Due to the large number (nearly 2000) of variables present in the database, conducting variable selection to select a subset of top predictors was necessary, and it could provide numerous advantages, such as reducing computer storage requirements, machine learning model training times, and data dimensionality, which might also lead to improved prediction performance [23]. The top predictor variables were chosen based on relative variable importance, using random forest. Random forest is a well-used tree method for variable selection. It works by identifying a smaller number of relevant predictors, resulting in a more parsimonious model, but with a similar predictive performance to a logistic model [24]. Using random forest, we identified the top 30 features (i.e., variables) ordered by their predictive performance. These 30 features include age, total hospital discharges, number of diagnoses, number of chronic conditions, length of stay, number of procedures, gender, discharged comorbidities (e.g., diabetes, hypertension, hypothyroidism, chronic obstructive pulmonary disease, renal failure, depression, peripheral vascular disorder, and obesity), hospital bed size, hospital type, discharge status. Age was based at the time of admission. The total number of hospital discharges was the sum of all the hospital discharges that the patient had experienced. The number of diagnoses was the total number of conditions that the patient had diagnosed. Similarly, the number of chronic conditions was the total number of chronic conditions that the patient had diagnosed. Length of stay was measured in days from the date of admission until the patient was discharged. Detail descriptions of all of these features can be found in Table 1 The 30 features were further narrowed down into a simpler model, with the top 6 features that showed relatively high variable importance, which were input into the machine learning classifiers.

### 2.6. Machine Learning Algorithims

In traditional statistical approaches, one must build a model, and then input the model into a machine (e.g., computer) [25]. This model-driven approach heavily relies on assumptions about the shape of the data, and may be prone to bias and error. Machine learning provides a data-driven approach to analyzing data. Instead of starting with an assumption about the data and the model, machine learning inputs the data directly into the machine. The goal of the machine is to perform pattern recognition in order to “learn” and output a model of the data [25]. Such an approach is particularly well-suited to analyzing large complex data, such as those of hospital readmissions data, genomic data, imaging data, or stock market data, where patterns are difficult to discern. Machine learning has great potential and implication in the public health spectrum for identifying healthcare needs, as well as crisis prediction and prevention [26].

For analysis, we classified the data using supervised machine learning approaches, including *k*-nearest neighbors (*k*-NN), support vector machine (SVM), and decision tree classifier. Supervised machine learning was chosen, because we already had the outcome of interest in mind (i.e., hospital readmission status of the patient) [27]. *K*-NN, SVM, and decision tree classifier are some of the most well-known and well-used methods to apply classification algorithms. Decision tree classifier provides advantages of efficiency and flexibility that might lead to performance improvements and is used in a wide array of areas such as medical diagnosis, remote sensing, and speech recognition [28]. *K*-NN is widely used for pattern classification, and is effective when the probability distribution of the input variables is unknown, as it does not make probability assumption of the variables [29]. SVM is well-suited for binary classification [30], and has been shown to work well with high dimensional data [31].

To account for overfitting, the data were randomly split into 60% training sets and 40% test sets. Models were then applied to both the training and test sets, and their accuracies were recorded. We aimed to keep the difference of the accuracies between the training and the test sets to be no more than 7%, to avoid overfitting of the data. We adjusted the model parameters when the data were overfitted.

This study was a secondary analysis of deidentified, publicly available data; thus, review from the institutional review board was exempted per US federal regulations (45 CFR 46, category 4).

## 3. Results

For the 30-day readmissions, there were a total of 11,334 cases (weighted *N* = 24,746) of AF patients undergoing catheter ablation. After applying diagnosis and procedural exclusion and accounting for index admissions and death, there were 5872 cases (weighted *N* = 12,634) remaining for data analysis. The 30-day readmission rate was 11.0%. The average age of the patients was 64.3 years old. Furthermore, 62.6% of the study participants were male (Table 2).

**Figure 1:**
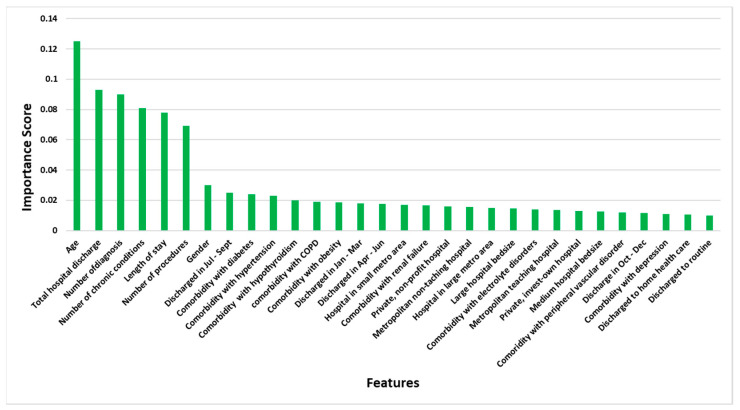
Relative variable importance of the top 30 features in predicting 30-day hospital readmissions in atrial fibrillation patients undergoing catheter ablation.

Random forest selected the top 30 features for determining the likelihood of a patient being readmitted for atrial fibrillation, with the patient’s age as the most important feature, since it has the highest importance score (Figure 1). The higher a variable’s importance score, the more useful or important the variable is at predicting hospital readmissions for atrial fibrillation. The top predictor variables identified for the 30-day readmissions were patient’s age, total discharges from a hospital, number of diagnoses a patient had on their discharge, number of chronic conditions a patient had on their discharge, number of procedures a patient had on their discharge, length of hospital stay, and gender.

**Figure 2:**
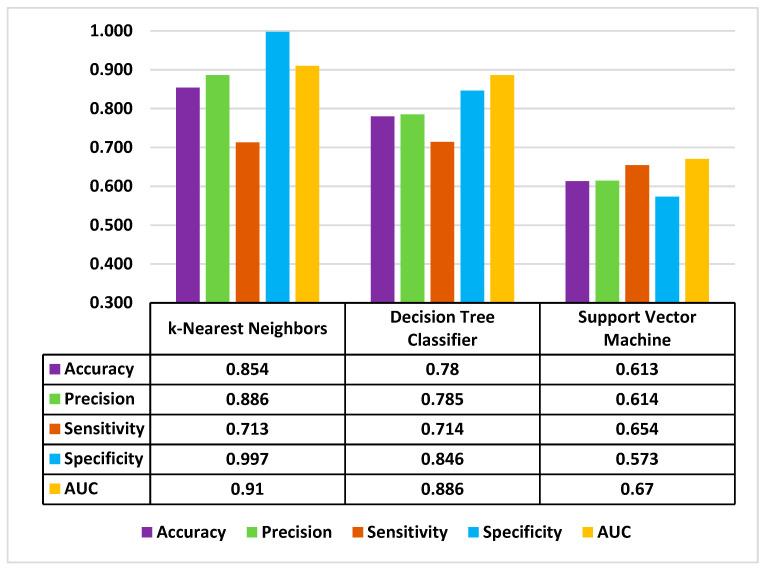
Performance metrics of machine learning models using the top 6 features (30-day readmissions).

**Figure 3:**
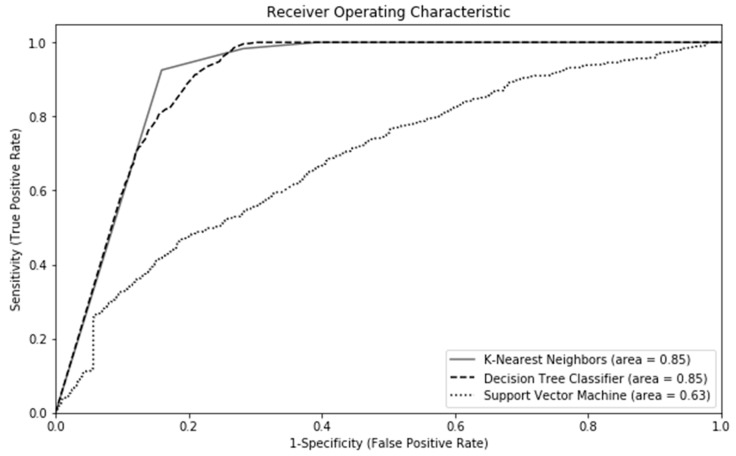
Area Under the Receiver Operating Characteristic curves for the various machine learning methods (30-day Readmissions).

Performance of machine learning classifiers can be described using accuracy, precision, sensitivity, specificity and area under the curve (AUC). Accuracy refers to the total number of correct predictions out of the total number of predictions made. Precision is the positive predictive value, measuring the proportion of positive cases identification that is actually correct. Sensitivity is the true positive rate, while specificity is the true negative rate. AUC is a metric for measuring the ability of a machine learning’s classifier to distinguish the two classes of outcomes (e.g., readmitted versus not readmitted). In general, one metric should be selected to evaluate the key performance of machine learning. We decided to use accuracy as the key performance indicator, as the total number of correct predictions out of all predictions was the interest of this study. Among the machine learning methods, *k*-NN had the highest accuracy at around 85%, followed by decision tree classifier at 78.0% (Figure 2). The SVM had the worst performance, at 61.3%. The performance indicators including accuracy, precision sensitivity, specificity, and AUC are displayed in Figure 2. The SVM had the worst performance metrics compared to the other two classifiers (Figure 3).

## 4. Discussion

This study aimed to predict 30-day readmissions status for AF patients undergoing catheter ablation. Our findings showed that machine learning models were able to accurately predict the occurrence of hospital readmissions at around 85% accuracy for the 30-day readmissions. The top predictors were: age, total discharges from hospital, number of diagnoses a patient had upon discharge, the number of chronic conditions a patient had upon discharge, the number of procedures a patient had on their record, length of hospital stay, and gender. Future research can consider the inclusion of additional variables beyond those in the NRD, to achieve a higher predictive accuracy.

One limitation of this study is the cross-sectional nature of the 2013 NRD data. Healthcare references and tools such as the ICD manuals have been updated since the collection of these data. Using data from multiple years would allow the development of potentially more accurate predictive models. Future studies may consider collecting longitudinal data to model prediction and confirm the results. Though hospital characteristics have been found to be largely influential factors in predicting readmissions for other conditions such as heart failure [32], this does not seem to be as important as a predictive factor for atrial fibrillation readmissions. It is important to note that translating these findings to institutional policies will be difficult for hospitals without the requisite budget. Readmissions prevention measures are more feasible for larger hospitals, with many beds, academic affiliations, adequate staffing, and a greater proportion of Medicare and privately insured patients. It may also be relatively easy to manage AF in outpatient settings compared to other cardiac conditions such as heart failure.

Previous research indicated that older age and various comorbidities of patients who underwent AF ablation are characteristics independently associated with an increased likelihood of readmissions, which corresponds with the findings of this study [33]. Specifically, patients with five or more comorbidities were twice as likely (or more) to be readmitted. Prior research had also identified gender, length of hospital stay, disposition to facility [22], as well as the number of chronic conditions [33], as the top predictors for 30-day readmissions, which was consistent with our findings. Our study was able to further identify additional top predictors (e.g., total number of discharges in hospital, number of diagnoses a patient had on their discharge, and number of procedures a patient had on their discharge), which were missed by previous research studies that used traditional statistical approaches. The discrepancies between our research and prior research might be attributed to the differences in analytical methods. Prior research had utilized mainly traditional statistical methods for analysis. Using machine learning to conduct analyses and build models can lead to an improved understanding of the data, and provide an innovative opportunity for new frontiers of discovery.

Using a supervised machine learning approach, our models were able to achieve a predictive accuracy of 85%. Such models can be valuable for policymakers and healthcare providers alike. Healthcare providers might find it useful to look closely into a patient’s record, and provide patients with more personalized medical treatments, to minimize hospital readmissions and improve healthcare quality. Applying predictive modeling to assess risks can result in effective preventative treatments, leading towards lower costs, improvement in care, and fewer mortalities [26].

## Figures and Tables

**Table 1:** Description of the top 30 features.

Variable	Description
Age	Age in years at admission
Total Hospital Discharges	Total number of hospital discharges the patient has experienced
Number of Diagnosis	Total number of diagnosed conditions for the patient
Number of Chronic Conditions	Total number of chronic conditions
Length of Stay	Length of stay (days)
Number of Procedures	Number of procedures on this discharge
Gender	Gender (male or female)
Discharged in Jul-Sept	Date of discharge was between July and September
Comorbidity with Diabetes	Patient has comorbidity with diagnosed diabetes
Comorbidity with Hypertension	Patient has comorbidity with diagnosed hypertension
Comorbidity with Hypothroidism	Patient has comorbidity with diagnosed hypothyroidism
Comorbidity with COPD	Patient has comorbidity with diagnosed COPD
Comorbidity with Obesity	Patient has comorbidity with diagnosed obesity
Discharged Jan-Mar	Date of discharge was between January and March
Discharged in Apr-Jun	Date of discharge was between April and June
Hospitality in Small Metro Area	Hospital is located in a small metro area
Comorbidity with Renal Failure	Patient has comorbidity with diagnosed renal failure
Private, Non-Profit Hospital	Hospital is categorized as a private, non-profit hospital
Metropolitan Non-teaching Hospital	Hospital is categorized as a metropolitan non-teaching hospital
Hospital in Large Metro Area	Hospital is located in a large metro area
Large Hospital Bedsize	Size of hospital beds is large
Comorbidity with Electrolyte Disorder	Patient has comorbidity with diagnosed electrolyte disorder
Metropolitan Teaching Hospital	Hospital is categorized as a metropolitan teaching hospital
Private, Invest-Own Hospital	Hospital is categorized as private, invest-own hospital
Medium Hospital Bedsize	Size of hospital beds is medium
Comorbidity with Peripheral Vascular Disorder	Patient has comorbidity with peripheral vascular disorder
Discharged in Oct-Dec	Date of discharge was between October and December
Comorbidity with Depression	Patient has comorbidity with diagnosed depression
Discharged to Health Home Care	Patient was discharged from hospital to go home health care.
Discharged to Routine	Patient was discharged from hospital to go home

**Table 2:** Demographic characteristics of 30-day Readmissions (Ninside of parenthesis is unweighted).

Variables	Mean	SD	Median	*N*	%
Age (years)	64.3(64.9)	11.6(11.4)	66(66)	12,634(5872)	100
Number of Chronic Conditions	5.2 (5.2)	2.7(2.7)	5(5)	12,634(5872)	100
Number of Diagnosis	8.2(8.1)	4.8(4.7)	7(7)	12,634(5872)	100
Number of Procedures	3.6(3.6)	1.6(1.6)	3(3)	12,634(5872)	100
Length of Stay (days)	2.5(2.4)	3.0(2.9)	1(1)	12,634(5872)	100
Gender					
Male				7906 (3652)	62.6(62.2)
Female				4728(2220)	37.4(37.8)
Income					
0–25th percentile				2453(1152)	19.7(19.9)
26th to 50th percentile				3040(1389)	24.4(24.0)
51st to 75th percentile				3308(1498)	26.6(25.9)
76th to 100th percentile				3650(1741)	29.3(30.1)
Expected Primary Payer					
Medicare				7029(3290)	55.6(56.0)
Medicaid				391(188)	3.1(3.2)
Private Insurance				4726(2201)	38.0(37.5)
Self-pay				77(40)	0.6(0.7)
No charge				26(13)	0.2(0.2)
Other				311(139)	2.5(2.4)
